# Tumor microenvironment-adjusted prognostic implications of the *KRAS* mutation subtype in patients with stage III colorectal cancer treated with adjuvant FOLFOX

**DOI:** 10.1038/s41598-021-94044-4

**Published:** 2021-07-16

**Authors:** Hye Eun Park, Seung-Yeon Yoo, Nam-Yun Cho, Jeong Mo Bae, Sae-Won Han, Hye Seung Lee, Kyu Joo Park, Tae-You Kim, Gyeong Hoon Kang

**Affiliations:** 1 Department of Pathology, Seoul National University Boramae Hospital, Seoul, South Korea; 2 Laboratory of Epigenetics, Cancer Research Institute, Seoul National University College of Medicine, Seoul, South Korea; 3 Department of Pathology, Asan Medical Center, University of Ulsan College of Medicine, Seoul, South Korea; 4 Department of Pathology, Seoul National University College of Medicine, Seoul National University Hospital, 103 Daehak-ro, Jongno-gu, Seoul, 03080 South Korea; 5 Department of Internal Medicine, Seoul National University Hospital, Seoul, South Korea; 6 Department of General Surgery, Seoul National University Hospital, Seoul, South Korea

**Keywords:** Colorectal cancer, Cancer, Gastroenterology, Oncology

## Abstract

Several studies have reported that the prognostic effect of *KRAS* mutations on colorectal cancers (CRCs) varies depending on the type of mutation. Considering the effect of *KRAS* mutations on tumor microenvironment, we analyzed the prognostic significance of *KRAS* mutation types after adjusting for the tumor-infiltrating lymphocytes (TIL) and tumor-stromal percentage (TSP) statuses. In two independent cohorts, *KRAS* mutations were analyzed by Sanger sequencing and/or next-generation sequencing. TIL density and the TSP were quantified from whole-slide immunohistochemical images. *KRAS*-mutant CRCs were divided into three subgroups (G12D/V, other codon 12 mutations and codon 13 mutations) to examine their differential effect on TIL density, the TSP and recurrence-free survival (RFS). Among the *KRAS* mutations, only the G12D/V subgroups showed significantly less TIL infiltration than the wild-type CRCs. According to survival analysis, G12D/V mutations were associated with short RFS; codon 13 mutations showed discordant trends in the two cohorts, and other codon 12 mutations showed no significant association. Multivariate analysis further supported the prognostic value of G12D/V mutations. This result is not only consistent with a recent study suggesting the immunosuppressive effect of mutant *KRAS* but also provides insight into the type-specific prognostic effect of *KRAS* mutations.

## Introduction

Colorectal cancer (CRC) is the third most common cancer in men and the second most common cancer in women worldwide, with an estimated incidence of 1.8 million cases in 2018^1^. Despite the improvement of survival due to the early detection of CRC and advances in systemic treatment, a quarter of patients present with metastatic disease, and the 5-year survival rate is less than 10% for patients with metastatic CRC^2–4^. CRCs develop through the progressive accumulation of genetic and epigenetic events along the adenoma-carcinoma sequence pathway or serrated neoplasia pathway^5^. Alterations in oncogenes and tumor suppressor genes are known to confer selective growth advantages, and the *KRAS* gene is mutated in over 40% of CRCs. Although the *KRAS* mutation activates the progrowth signaling pathway, recent studies have indicated that the *KRAS* mutation contributes to immune suppression for the evasion of tumor cells from the host immune response^6^.

The *KRAS* gene encodes guanosine triphosphate hydrolase (GTPase), which is involved in epidermal growth factor receptor (EGFR) signaling. RAS proteins normally transmit or block signal transduction by regulating an active GTP binding form and an inactive GDP binding form. However, *KRAS* mutations result in the GTP-binding form of the RAS protein, which is constantly activated due to deficiency in GTPase activity, and the signaling pathway is permanently activated without the upstream stimulation of EGFR. Thus, *KRAS* mutations are known as predictive markers for resistance to anti-EGFR therapies such as cetuximab or panitumumab^7^. However, the prognostic value of the *KRAS* mutation is still unclear^8^. One large-scale study of patients with stage III CRC reported that *KRAS* mutations were associated with poor survival in microsatellite-stable (MSS) CRC but not in microsatellite-unstable CRC^9^. Most somatic missense mutations in the *KRAS* gene occur in codon 12, followed by codon 13, while mutations in codon 61 or 146 are rarely reported^10^. Several studies have shown that patient outcomes depend on specific *KRAS* mutations, but the results have been inconsistent^9, 11^.

Recently, several studies have demonstrated that *KRAS* mutations regulate the tumor microenvironment in various cancer types^6^. One in vivo study showed that granulocyte macrophage colony-stimulating factor (GM-CSF) from *Kras*-mutant cells promotes the recruitment of myeloid-derived suppressor cells (MDSCs), which inhibit the antitumor activity of CD8 + cytotoxic T cells in a mouse model of pancreatic cancer with *Kras* and *Trp53* mutations^12^. A similar effect has been reported in human CRCs, in which elevated levels of specific interleukins and GM-CSF expression were associated with *KRAS* mutations^13^. An experimental study using the *Kras*/*Apc*/*Trp53* mouse model and human CRC tissues demonstrated that oncogenic *Kras* promoted MDSC migration and decreased T cell infiltration into the CRC microenvironment by inhibiting interferon regulatory factor 2 (IRF2) expression and subsequently activating *Cxcl3*^14^. In addition, it was reported that specific immune subpopulations, such as cytotoxic T cells and neutrophils, and the IFN gamma pathway were suppressed in *KRAS*-mutant CRCs^15^. However, we found that no study has analyzed a difference in the density of TILs according to specific *KRAS* mutations.

A close relationship between the *KRAS* mutation and the amount of cancer stroma (tumor stromal percentage, TSP) has also been suggested. *KRAS* mutations can affect Notch1 signaling and epithelial-mesenchymal transition by inducing overexpression of Jagged1^16^. In genetically engineered mouse models of CRC, activated Notch1 signaling upregulates *Tgfb2* expression in cancer cells and *Tgfb1* expression in stomal cells^17^. *KRAS* mutations are known to extend their oncogenic signaling beyond cancer cells to cancer-associated fibroblasts^18^ and promote their migration via various signals^19^.

Although researchers have analyzed the prognostic values of different *KRAS* mutant types in patients with CRC^9, 20^, little is known regarding the difference in TIL density and the TSP according to the *KRAS* mutant type. In a previous study, TIL density and the TSP were computationally quantified using whole-slide images of CD3 and CD8 immunohistochemical stains^21^. In the present study, based on our speculation that CRCs may harbor different TIL densities and TSPs depending on the *KRAS* mutant type, we investigated the difference in TIL density and the TSP of CRCs according to *KRAS* mutant types and attempted to identify whether there is a difference in prognosis by adjusting for TIL density and the TSP. We analyzed *KRAS* mutations in two cohorts of stage III CRC patients who received adjuvant FOLFOX. We demonstrate that specific *KRAS* mutation types, not all *KRAS* mutation types, are associated with a low density of TILs and that the *KRAS* mutation subtype is an independent prognostic parameter in patients with stage III CRC treated with oxaliplatin-based adjuvant chemotherapy.

## Results

**Figure 1:**
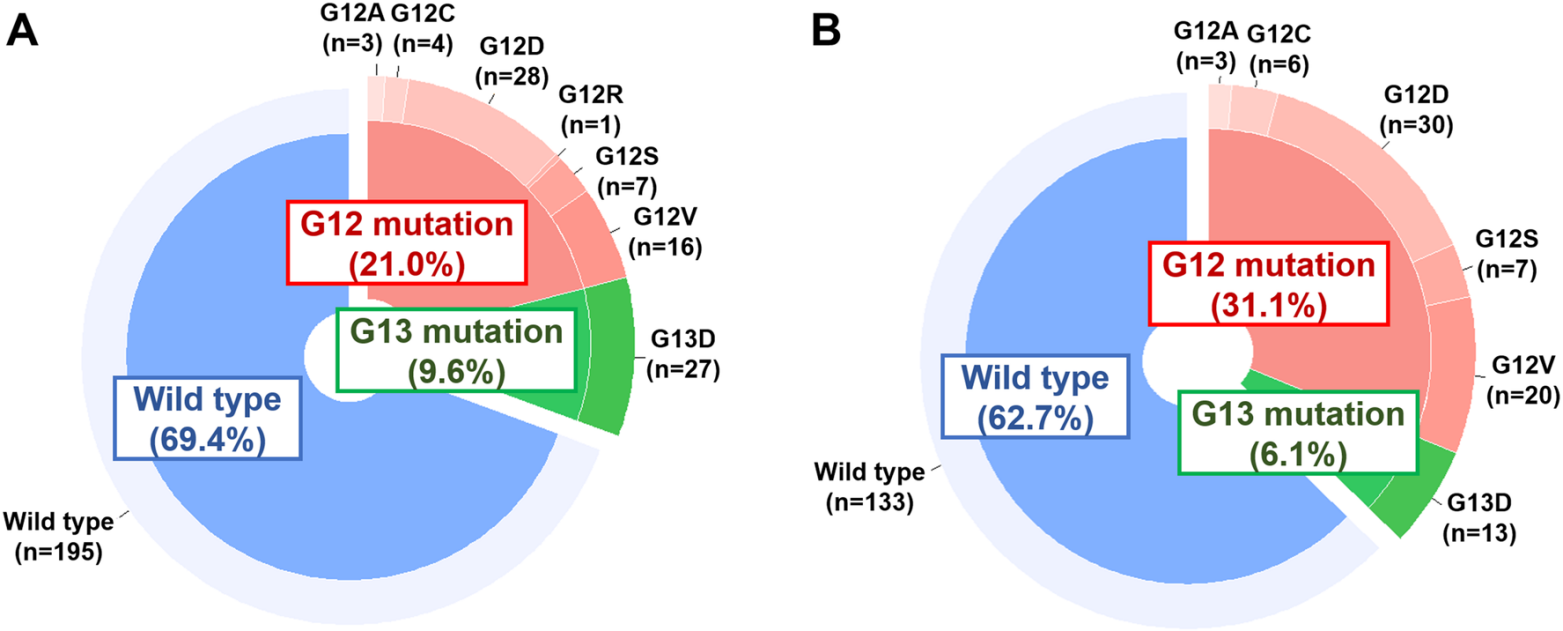
Distribution of *KRAS*-mutant and wild-type statuses in the (**A**) discovery cohort and (**B**) validation cohort.

*KRAS* mutations were identified in 30.6% (n = 86) of patients in the discovery cohort and 37.3% (n = 79) of patients in the validation cohort. Among the CRCs with *KRAS* mutations, codon 12 mutations accounted for 68.6% (n = 59) of those in the discovery cohort and 83.5% (n = 66) of those in the validation cohort. The majority of codon 12 mutations were G12D and G12V mutations (n = 44 in the discovery cohort; n = 50 in the validation cohort), followed by G12S, G12C, G12A, and G12R, while codon 13 mutations were represented only by G13D (Fig. 1).

### Association of the *KRAS* mutation type with TIL density and the TSP

**Figure 2:**
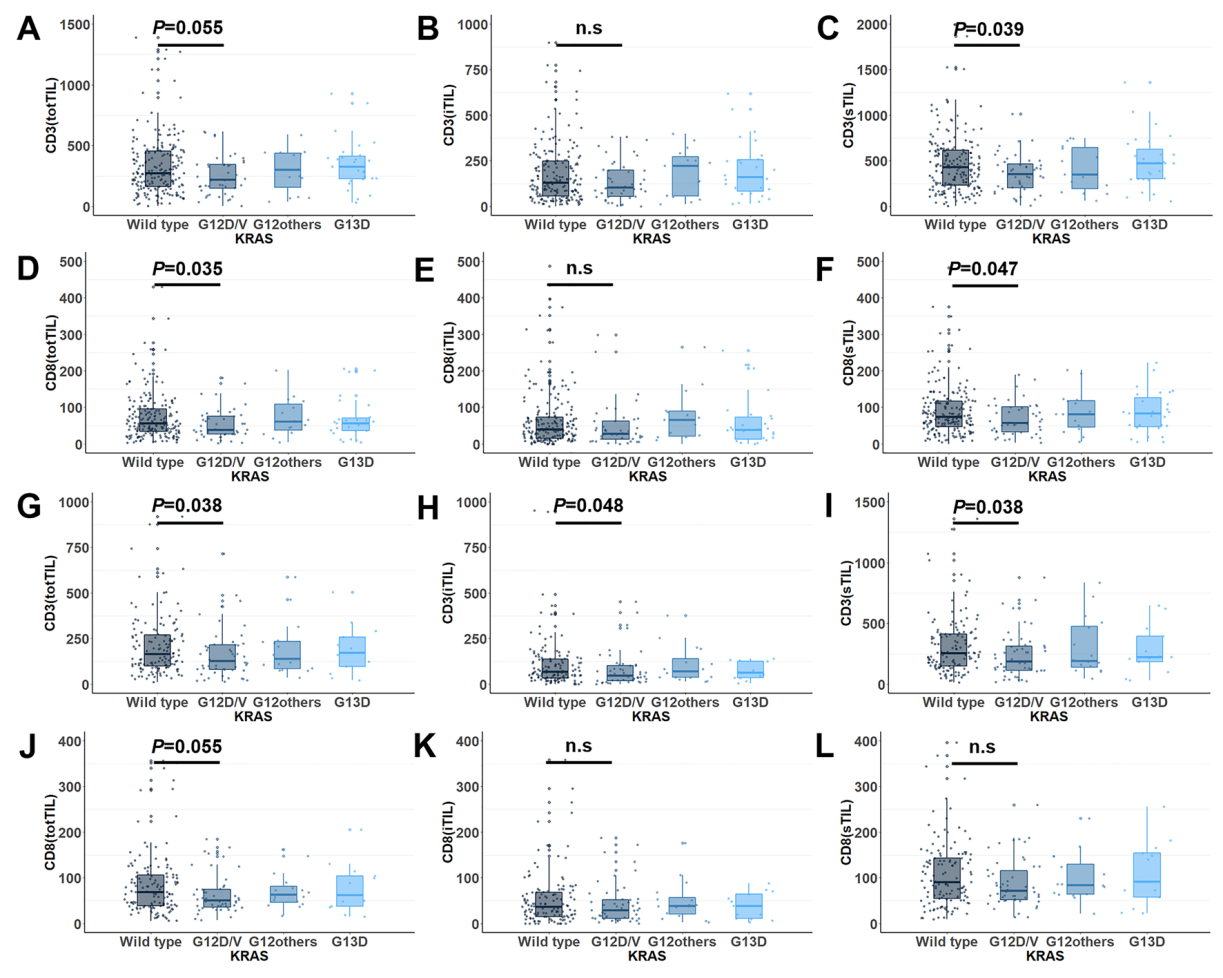
Density of tumor-infiltrating lymphocytes according to the *KRAS* mutation status in the (**A**–**F**) discovery cohort and (**G**–**L**) validation cohort.

**Figure 3:**
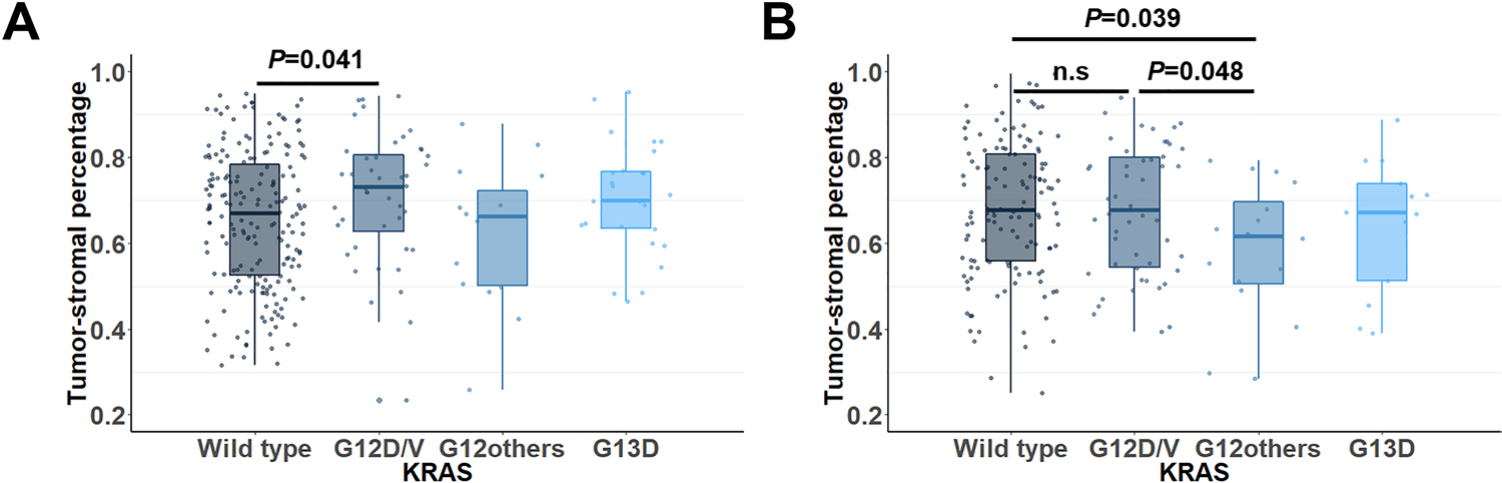
Tumor-stromal percentage according to the *KRAS* mutation status in the (**A**) discovery cohort and (**B**) validation cohort.

When comparing clinicopathologic parameters according to the *KRAS* mutation status, no significant difference was found between CRCs with wild-type *KRAS* and CRCs with mutant *KRAS* except for the density of TILs (Table 1 and Fig. 2). The association between *KRAS* mutations and TILs was identified by comparisons of CD3(totTILs), CD3(iTILs), CD3(sTILs), CD8(totTILs), CD8(iTILs), and CD8(sTILs) according to specific *KRAS* mutation types. *KRAS*-mutant CRCs were divided into three subgroups: G12D/V, other codon 12 mutation types, and the codon 13 mutation (G13D). The subgroup analysis showed that the TIL density of CRCs with G12D/V was significantly lower than that of wild-type CRCs, and a significant decrease in CD3(sTILs) was observed in both the discovery and validation cohorts. The lower density of CD8(totTILs) and CD8(sTILs) in the G12D/V group was statistically significant but only in the discovery cohort, not in the validation cohort. CD3(totTILs) and CD3(iTILs) tended to decrease in the G12D/V group; although the difference was not statistically significant in the discovery cohort, the difference was statistically significant in the validation cohort. In the discovery cohort, the TSP was significantly different between CRCs with wild-type *KRAS* and CRCs with the *KRAS* G12D/V mutant (Fig. 3A). However, the validation cohort did not show such a relationship between the TSP and *KRAS* mutation type (Fig. 3B).

**Table 1:** Comparison of clinicopathologic parameters between colorectal cancers with wild-type and mutant *KRAS* in the discovery and validation cohorts.

Factor	Discovery cohort			Validation cohort		
	Wild-type *KRAS* (n = 195)	Mutant *KRAS* (n = 86)	*p* value	Wild-type *KRAS* (n = 133)	Mutant *KRAS* (n = 79)	*p* value
Age (y)	59.8 ± 8.70	59.7 ± 9.77	0.930	60.7 ± 9.93	60.7 ± 10.39	0.860
**Sex**			0.325			0.513
Male	121 (62.1%)	48 (55.8%)		73 (54.9%)	47 (59.5%)	
Female	74 (37.9%)	38 (44.2%)		60 (45.1%)	32 (40.5%)	
**Location**			0.165			0.476
Right-sided	50 (25.6%)	29 (33.7%)		49 (36.8%)	33 (41.8%)	
Left-sided	145 (74.4%)	57 (66.3%)		84 (63.2%)	46 (58.2%)	
**T stage**			0.425			0.001
T1-3	173 (88.7%)	79 (91.9%)		114 (85.7%)	52 (65.8%)	
T4	22 (11.3%)	7 (8.1%)		19 (14.3%)	27 (34.2%)	
**N stage**			0.915			0.057
N1	126 (64.6%)	55 (64.0%)		96 (72.2%)	47 (59.5%)	
N2	69 (35.4%)	31 (36.0%)		37 (27.8%)	32 (40.5%)	
**Tumor differentiation**			0.833			0.021
Well-moderately	180 (92.3%)	80 (93.0%)		105 (78.9%)	72 (91.1%)	
Poorly	15 (7.7%)	6 (7.0%)		28 (21.1%)	7 (8.9%)	
**Mucin**			0.079			0.137
Absent	184 (94.4%)	76 (88.4%)		112 (84.2%)	60 (75.9%)	
Present	11 (5.6%)	10 (11.6%)		21 (15.8%)	19 (24.1%)	
**Lymphatic invasion**			0.593			0.142
Absent	111 (56.9%)	46 (53.5%)		66 (49.6%)	31 (39.2%)	
Present	84 (43.1%)	40 (46.5%)		67 (50.4%)	48 (60.8%)	
**Venous invasion**			0.780			0.208
Absent	170 (87.2%)	76 (88.4%)		106 (79.7%)	57 (72.2%)	
Present	25 (12.8%)	10 (11.6%)		27 (20.3%)	22 (27.8%)	
**Perineural invasion**			0.528			0.584
Absent	141 (72.3%)	59 (68.6%)		74 (55.6%)	47 (59.5%)	
Present	54 (27.7%)	27 (31.4%)		59 (44.4%)	32 (40.5%)	
MSI*			0.783			0.014
MSS/MSI-L	183 (93.8%)	81 (95.3%)		111 (87.4%)	76 (97.4%)	
MSI-H	12 (6.2%)	4 (4.7%)		16 (12.6%)	2 (2.6%)	
***BRAF***			0.035			0.015
Wild type	185 (94.9%)	86 (100.0%)		123 (92.5%)	79 (100.0%)	
Mutated	10 (5.1%)	0 (0.0%)		10 (7.5%)	0 (0.0%)	
CIMP**			0.085			0.089
CIMP-N	180 (92.3%)	78 (91.8%)		115 (86.5%)	76 (96.2%)	
CIMP-P1	8 (4.1%)	7 (8.2%)		11 (8.3%)	2 (2.5%)	
CIMP-P2	7 (3.6%)	0 (0.0%)		7 (5.3%)	1 (1.3%)	

*MSI status was determined in 280 patients in the discovery cohort and 205 patients in the validation cohort.

**CIMP status was not analyzed in one patient in the discovery cohort.

### Survival analysis of patients with stage III CRC according to the *KRAS* mutation type

**Figure 4:**
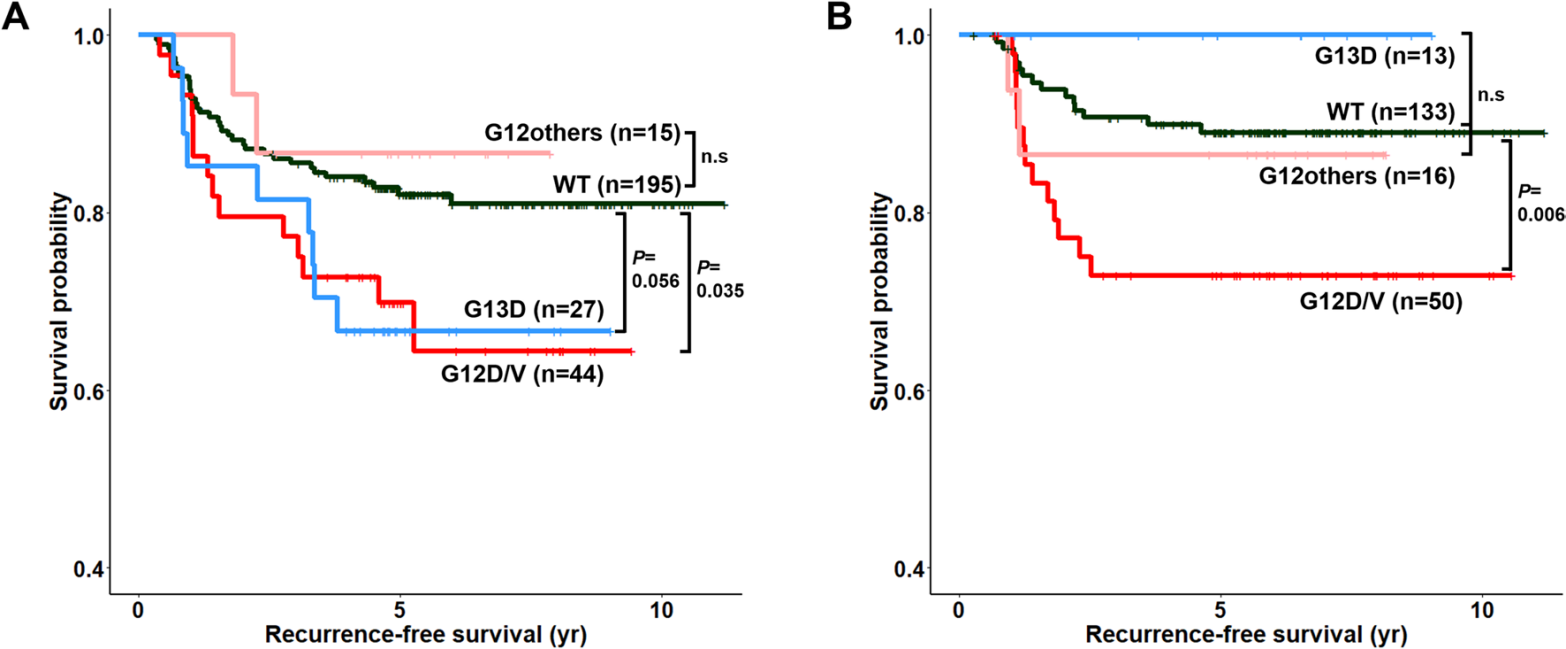
Recurrence-free survival according to the *KRAS* mutation status. Kaplan–Meier survival curve of the (**A**) discovery cohort and (**B**) validation cohort.

In the Kaplan–Meier survival analysis, G12D/V mutations were associated with poor RFS in both cohorts, while there was no difference in survival between the other G12 mutations and wild-type CRCs (*P* = 0.035 in the discovery cohort; *P* = 0.006 in the validation cohort, Fig. 4). The G13D mutation was associated with inferior outcomes in the discovery cohort (*P* = 0.056) but not in the validation cohort, showing inconsistent results. To further demonstrate the value of the *KRAS* mutation subtype as an independent prognostic factor, we performed multivariate Cox proportional hazards analysis in the discovery and validation cohorts (Tables 2, 3). Additionally, T category, N category, tumor differentiation, lymphovascular emboli, perineural invasion, CD8(iTILs) and the TSP were included in the multivariate analysis because CD8(iTILs) and the TSP were found to be significant prognostic factors in the univariate survival analysis. Of the four TIL parameters, CD8(iTILs) showed the highest prognostic significance in the univariate analysis; thus, the CD8(iTILs) parameter was included in the multivariate analysis (Supplementary Table 1). On the multivariate analysis, the G12D/V mutation was associated with a poor prognosis in both cohorts, with marginal significance (HR = 2.015, 95% CI = 1.064–3.817, *P* = 0.032 in the discovery cohort; HR = 2.366, 95% CI = 1.109–5.046, *P* = 0.026 in the validation cohort). The G13D mutation demonstrated a significant effect on survival in the discovery cohort but not in the validation cohort (HR = 3.041, 95% CI = 1.411–6.554, *P* = 0.005 in the discovery cohort; HR = 0.000, 95% CI = 0.000-Inf, *P* = 0.997 in the validation cohort).

**Table 2:** Univariate and multivariate analyses in the discovery cohort.

		Univariate analysis		Multivariate analysis	
		HR (95% CI)	*p* value	HR (95% CI)	*p* value
**Location**					
Right	79	1 (ref)			
Left	202	0.875 (0.504–1.521)	0.637		
**Tumor differentiation**					
Well-moderately	260	1 (ref)		1 (ref)	
Poorly	21	3.504 (1.821–6.744)	< 0.001	3.479 (1.749–6.919)	< 0.001
**T stage**					
T1-3	252	1 (ref)		1 (ref)	
T4	29	3.054 (1.651–5.649)	< 0.001	2.504 (1.304–4.809)	0.006
**N stage**					
N1	181	1 (ref)		1 (ref)	
N2	100	2.652 (1.576–4.377)	< 0.001	1.902 (1.125–3.214)	0.016
**Lymphovascular emboli**					
Absent	148	1 (ref)		1 (ref)	
Present	133	2.998 (1.725–5.210)	< 0.001	2.338 (1.320–4.139)	0.004
**Perineural invasion**					
Absent	200	1 (ref)		1 (ref)	
Present	81	2.159 (1.294–3.601)	0.003	1.484 (0.866–2.543)	0.151
**KRAS**					
Wild type	195	1 (ref)		1 (ref)	
G12D/V mutations	44	1.925 (1.035–3.580)	0.038	2.015 (1.064–3.817)	0.032
G12 other mutations	15	0.718 (0.173–2.986)	0.649	1.062 (0.251–4.489)	0.935
G13D mutation	27	2.020 (0.970–4.207)	0.060	3.041 (1.411–6.554)	0.005
MSI*					
MSS/MSI-L	264	1 (ref)			
MSI-H	16	0.925 (0.290–2.953)	0.895		
Tumor-stromal percentage**					
Low	210	1 (ref)		1 (ref)	
High	71	2.344 (1.397–3.934)	0.001	2.036 (1.192–3.477)	0.009
**CD8(iTILs)**					
Low	141	1 (ref)		1 (ref)	
High	140	0.265 (0.146–0.483)	< 0.001	0.287 (0.155–0.530)	< 0.001

*MSI status was not analyzed in one patient.

**CRCs were divided into a high group (upper one quartile of the tumor-stromal percentage) and a low group (lower three quartiles of the tumor-stromal percentage).

Multivariate analysis was performed without inclusion of the CIMP.

**Table 3:** Univariate and multivariate analyses in the validation cohort.

		Univariate analysis		Multivariate analysis	
		HR (95% CI)	*p* value	HR (95% CI)	*p* value
**Location**					
Right	82	1 (ref)			
Left	130	0.650 (0.314–1.347)	0.246		
**Tumor differentiation**					
Well-moderately	177	1 (ref)			
Poorly	35	1.102 (0.420–2.887)	0.844		
**T stage**					
T1-3	166	1 (ref)			
T4	46	1.701 (0.774–3.736)	0.186		
**N stage**					
N1	143	1 (ref)		1 (ref)	
N2	69	3.958 (1.868–8.383)	< 0.001	3.274 (1.531–7.000)	0.002
**Lymphovascular emboli**					
Absent	75	1 (ref)			
Present	137	1.457 (0.645–3.290)	0.365		
**Perineural invasion**					
Absent	121	1 (ref)			
Present	91	1.660 (0.799–3.452)	0.175		
***KRAS***					
Wild type	133	1 (ref)		1 (ref)	
G12D/V mutations	50	2.758 (1.296–5.870)	0.008	2.366 (1.109–5.046)	0.026
G12 other mutations	16	1.374 (0.312–6.049)	0.674	1.776 (0.374–8.436)	0.470
G13D mutation	13	4.491e−08 (0.000-Inf)	0.997	3.807e − 08 (0.000-Inf)	0.997
MSI*					
MSS/MSI-L	187	1 (ref)			
MSI-H	18	0.377 (0.051–2.781)	0.339		
Tumor-stromal percentage**					
Low	151	1 (ref)		1 (ref)	
High	61	3.461 (1.664–7.199)	0.001	2.467 (1.119–5.438)	0.025
**CD8(iTILs)**					
Low	105	1 (ref)		1 (ref)	
High	107	0.289 (0.124–0.677)	0.004	0.391 (0.163–0.937)	0.035

*MSI status was determined in 205 patients with CRC.

**CRCs were divided into a high group (upper one quartile of the tumor stromal percentage) and a low group (lower three quartiles of the tumor-stromal percentage).

## Discussion

*KRAS* mutations are common mutations in CRC and are well known as predictive markers for cetuximab therapy, but previous studies on their prognostic value have shown inconsistent results. In the present study, the *KRAS* G12D/V mutation was associated with a low TIL density and a poor prognosis in stage III CRC patients treated with adjuvant FOLFOX chemotherapy. To the best of our knowledge, this is the first study to validate the association between specific *KRAS* mutations and TIL density, which was quantified through whole-slide images and computer-based methods^21^. Although the association between a low TIL density and the *KRAS* G12D/V mutation raised concern over whether the association between a poor prognosis and the *KRAS* G12D/V mutation might be related to the low density of TILs, the multivariate analysis showed that both the *KRAS* G12D/V mutation and low TIL density were independent prognostic markers for a poor prognosis.

The frequency of *KRAS* mutations was 30–40% in the present study (30.6% in the discovery cohort and 37.3% in the validation cohort). The majority of *KRAS* mutations consisted of G12D, G12V, and G13D mutations, followed by G12A, G12C, G12S, and G12R. The Cancer Genome Atlas (TCGA) dataset also showed that KRAS mutations were identified in 40.8% of CRC patients (218 out of 534), with G12D (n = 58) being the most common mutation subtype, followed by G12V (n = 49) and G13D (n = 37)^22^. Several studies have reported differences in the survival of CRC patients according to specific subtypes of *KRAS* mutations. In a large-scale study with stage III CRC patients, *KRAS* mutations were associated with poor outcome in MSS/MSI-L CRC but not in MSI-H CRC^9^. In MSS/MSI-L CRCs, all codon 12 mutations and G13D mutations were associated with a shorter time to recurrence and overall survival. By contrast, Jones et al. reported that codon 12 mutations were an independent prognostic factor, but codon 13 mutations were not associated with poor overall survival (OS). In particular, both G12V and G12C mutations were associated with poor OS^23^. Similar to these results, Margonis et al. demonstrated that G12V and G12S mutations were independent prognostic factors of poor OS. Additionally, G12V, G12C and G12S mutations were associated with a poor prognosis in patients who experienced tumor recurrence after the resection of CRC liver metastasis^11^. Bai et al. suggested that codon 12 mutations, in particular, G12D and G12V mutations, were associated with a poor prognosis in Chinese patients with metastatic CRC^24^. In the present study, we divided *KRAS*-mutant CRCs into three subgroups, taking into account previous studies on the mutation rate, prognosis, and relative affinity for RAF kinase of individual *KRAS* mutation types (G12D/V, other codon 12 mutations, and the codon 13 mutation (G13D))^25, 26^. G12D/V mutations were consistently associated with a poor prognosis in both the discovery and validation cohorts, while the results regarding the effect of the codon 13 mutation on RFS were inconsistent. The subgroup of other codon 12 mutations showed no significant association with survival compared to wild-type CRCs.

Several studies have suggested that oncogenic potential or aggressiveness may differ according to specific *KRAS* mutations. A previous study demonstrated that codon 12 mutations were associated with resistance to apoptosis and aggressiveness (compared to codon 13 mutations)^27^. Al-Mulla et al. reported the possibility that G12V generates more persistent and potentially oncogenic signals than G12D due to the differences in GTPase activity and affinity for GTP^28^. We focused on the association with TIL density to elucidate the prognostic value of specific *KRAS* mutation types. Recently, it was recognized that mutant *KRAS* regulates tumor-associated immune responses and induces the protumorigenic properties of immune cells^29^. Lal et al. showed the reduced infiltration of cytotoxic T cells and downregulation of the IFNγ pathway in *KRAS*-mutant CRC^15^, and Liao et al. found that *KRAS* mutations induced an immune-suppressive profile by inhibiting IRF2 expression and promoting the migration of MDSCs^14^. In the present study, we demonstrated the association between specific *KRAS* mutations and the density of TILs by whole-slide image analysis in a homogeneous cohort of stage III CRC patients who received adjuvant FOLFOX chemotherapy. G12D/V mutations were consistently associated with a low TIL density and poor outcomes. Considering the effect of MSI on the tumor microenvironment, we analyzed the TIL density according to the *KRAS* mutations in MSS/MSI-L or MSI-H CRCs. The TIL density of CRCs with G12D/V mutations was still significantly lower than that of the wild-type CRCs in the MSS/MSI-L CRCs of the discovery cohort, and a similar tendency was observed in the validation cohort with no statistical significance (Supplementary Fig. 1). The MSI-H group failed to show a significant difference due to the limitation of the number of patients in both cohorts (n = 16 in the discovery cohort; n = 18 in the validation cohort).

Galon et al. designed a scoring system, “Immunoscore,” based on the quantification of cytotoxic and memory T lymphocytes or CD3 and CD8-positive T lymphocytes in the center and at the invasive margin of primary tumors^30, 31^. Immunoscore was shown to be a powerful prognostic factor in CRCs^32, 33^, and superior to MSI in predicting recurrence and survival^34^. Both *KRAS* mutations and Immunoscore were demonstrated as independent predictors of survival in the studies exploring the prognostic value of Immunoscore in CRCs^35, 36^, which is consistent with the results of the present study: both CD8(iTILs) and *KRAS* G12D/V mutations were found to be independent parameters in multivariate survival analysis. However, to the best of our knowledge, there is no study analyzing the difference of Immunoscore in CRCs according to *KRAS* mutation subtypes.

The strengths of this study include a well-defined cohort with stage III CRC treated with curative surgery and adjuvant FOLFOX. It also provides accurate *KRAS* mutation statuses confirmed by both direct sequencing and targeted NGS and computer-based quantification of TIL density by whole-slide image analysis. However, there are some limitations. First, rare mutation types such as mutations in codon 61 or 146 were excluded from this study because direct sequencing was performed only in *KRAS* exon 2 and not full-length *KRAS*. Second, the number of patients with each individual mutation type was very small, making it difficult to establish statistical significance. It is necessary to verify these results in larger cohorts and elucidate the mechanism how the *KRAS* mutation affects the infiltration of various immune cells in the tumor microenvironment. Third, the lack of assessment of *BRAF* and *NRAS* mutations which may pollute the *KRAS* wild-type group, is likely to lead to an insufficient power for the multivariate analysis. The effects of *NRAS*/*BRAF* mutation on the tumor microenvironment need to be evaluated in future studies using larger cohorts.

In conclusion, the findings of the present study indicate that among three types of *KRAS* mutations, G12D/V mutations were consistently associated with less TIL infiltration and shorter RFS in two independent cohorts of stage III CRC patients treated with adjuvant FOLFOX. This finding is not only consistent with a recent study suggesting the immunosuppressive effect of mutant *KRAS* but also provides insight into the type-specific prognostic effect of *KRAS* mutations.

## Materials and methods

### Patients and samples

The discovery and validation cohorts consisted of stage III CRC patients who had undergone curative surgery and received oxaliplatin-based adjuvant chemotherapy; the inclusion and exclusion criteria for these patients were described in detail previously^21, 37^. Among the cohort of patients with stage III CRC (n = 546) who had completed 6 or more cycles of adjuvant FOLFOX chemotherapy after curative surgery in Seoul National University Hospital (SNUH), Seoul, Korea, between April 2005 and December 2012, the discovery cohort included 281 patients with consistent *KRAS* mutation data from both targeted next-generation sequencing (NGS) and direct Sanger sequencing. The validation cohort consisted of 212 patients with stage III CRC who had received adjuvant FOLFOX after complete resection of the tumor at Seoul National University Bundang Hospital (SNUBH), Seongnam, Korea, between January 2007 and December 2012. The *KRAS* mutation status was evaluated by direct Sanger sequencing only. Clinical and histopathologic data were collected through electronic medical records and microscopic examinations. The clinicopathologic data included patient age, sex, recurrence-free survival (RFS), tumor location, American Joint Committee on Cancer (AJCC)/Union for International Cancer Control (UICC) tumor-node-metastasis (TNM) stage, tumor differentiation, lymphovascular invasion, and perineural invasion. This study was approved by the Institutional Review Board (IRB) of both SNUH (IRB No. 1811–061-983) and SNUBH (IRB No. B-1611/369–304).

### *KRAS* mutation analysis

Targeted NGS of 40 genes, including *KRAS*, was performed as described previously^38^. For Sanger sequencing of *KRAS*, representative tumor portions were marked histologically, and the corresponding areas on unstained tissue slides were then subjected to manual microdissection. The dissected tissues were collected into microtubes containing lysis buffer and proteinase K and incubated at 55 °C for up to 2 days. The direct sequencing of *KRAS* exon 2 was performed to confirm the NGS results. A total of 281 samples that had consistent results between NGS and Sanger sequencing were included in the discovery cohort. In the validation cohort, mutations in *KRAS* exon 2 were analyzed by direct sequencing only.

### Quantification of TIL density and the TSP from whole-slide immunohistochemical images

For each case, immunohistochemistry for CD3 and CD8 was performed on a representative tumor section, and the stains were subjected to computational quantification of TIL density and the TSP as described previously^21^. Briefly, stained slides were scanned on an Aperio AT2 slide scanner (Leica Biosystems) at 20 × magnification, and the virtual slide files were input into an analytic pipeline whose detailed protocol is available at http://dx.doi.org/10.17504/protocols.io.yqvfvw6. Once a user designated the tumor area of a given image, the algorithm segmented the area into 1 mm × 1 mm tiles and computed the median density (number of cells/mm^2^) of total TILs (totTILs), intraepithelial TILs (iTILs) and stromal TILs (sTILs) and the median TSP (stroma area/(tumor cell area + stroma area) X 100). As a consequence, the TSP and the following six TIL parameters were obtained from representative images of CD3 and CD8 immunohistochemical stains for each patient: CD3-positive totTILs (CD3(totTILs)), CD3-positive iTILs (CD3(iTILs)), CD3-positive sTILs (CD3(sTILs)), CD8-positive totTILs (CD8(totTILs)), CD8-positive iTILs (CD8(iTILs)), and CD8-positive sTILs (CD8(sTILs)).

### Analysis of microsatellite instability and the CpG island methylator phenotype

The microsatellite instability (MSI) status was determined through the evaluation of five microsatellite markers (BAT25, BAT26, D2S123, D5S346 and D17S250) as standardized by the National Cancer Institute. An MSI-high (MSI-H) status was defined as when tumor DNA had altered alleles in two or more markers compared to normal DNA. An MSI-low (MSI-L) status was defined as when tumor DNA had altered alleles in one marker compared to normal DNA. Microsatellite-stable (MSS) was defined as when no altered allele was present in tumor DNA. The status of the CpG island methylator phenotype (CIMP) was evaluated by a real-time methylation-specific qPCR method (MethyLight) and eight CIMP-specific markers (*CACNA1G*, *CDKN2A*, *CRABP1*, *IGF2*, *MLH1*, *NEUROG1*, *RUNX3*, and *SOCS1*). Tumors were classified as CIMP-negative, CIMP-P1, or CIMP-P2 when ≤ 4, 5–6, and ≥ 7 markers were methylated, respectively, as described previously^37^.

### Statistical analysis

In this study, statistical analysis was performed using SPSS version 25 (IBM, Armonk, NY, USA). Comparisons between categorical variables were conducted with the chi-square test or Fisher’s exact test. To determine whether TIL densities and the TSP were normally distributed, a normality test was performed with Shapiro–Wilk’s W test. TIL densities were not normally distributed, while the TSP was normally distributed. Because of these findings, both ANOVA and the Kruskal–Wallis test were performed to identify any difference in the means of parametric and nonparametric tests, respectively, between three or more groups. Student’s t test and the Mann–Whitney test were used for the comparison of the means of parametric and nonparameteric tests between two groups, respectively. Survival analysis was performed using the Kaplan–Meier method with the log-rank test. Hazard ratios (HRs) were calculated using the Cox proportional hazards model. All variables that were associated with RFS with *P* < 0.10 were entered into the model. These variables were reduced by backward elimination. All statistical tests were two-sided, and *P* < 0.05 was considered statistically significant.

### Ethics approval and consent to participate

All patients gave informed consent prior to specimen collection according to our institutional guidelines. The institutional review board of Seoul National University Hospital and Seoul National University Bundang Hospital approved this study. This study was performed in accordance with the Declaration of Helsinki.

## Supplementary Information

Supplementary Figure Legend.

Supplementary Figure 1.

Supplementary Table 1.

## Data Availability

The data sets used and/or analyzed during this study are available from the corresponding author on reasonable request.
