# Supplementary material for: Tumor microenvironment-adjusted prognostic implications of the KRAS mutation subtype in patients with stage III colorectal cancer treated with adjuvant FOLFOX
Source: Sci Rep. 2021 Jul 16;11:14609. doi: 10.1038/s41598-021-94044-4 (PMC8285533; doi:10.1038/s41598-021-94044-4)
Supplement: Supplementary file 1 — Supplementary Figure Legend. [file 41598_2021_94044_MOESM1_ESM.docx]

Supplementary Figure 1. Density of tumor-infiltrating lymphocytes according to the KRAS mutation status in MSS/MSI-L CRCs of the (a-f) discovery cohort and (g-l) validation cohort
