# Supplementary figures and images for: Tumor microenvironment-adjusted prognostic implications of the KRAS mutation subtype in patients with stage III colorectal cancer treated with adjuvant FOLFOX
Source: Sci Rep. 2021 Jul 16;11:14609. doi: 10.1038/s41598-021-94044-4 (PMC8285533; doi:10.1038/s41598-021-94044-4)

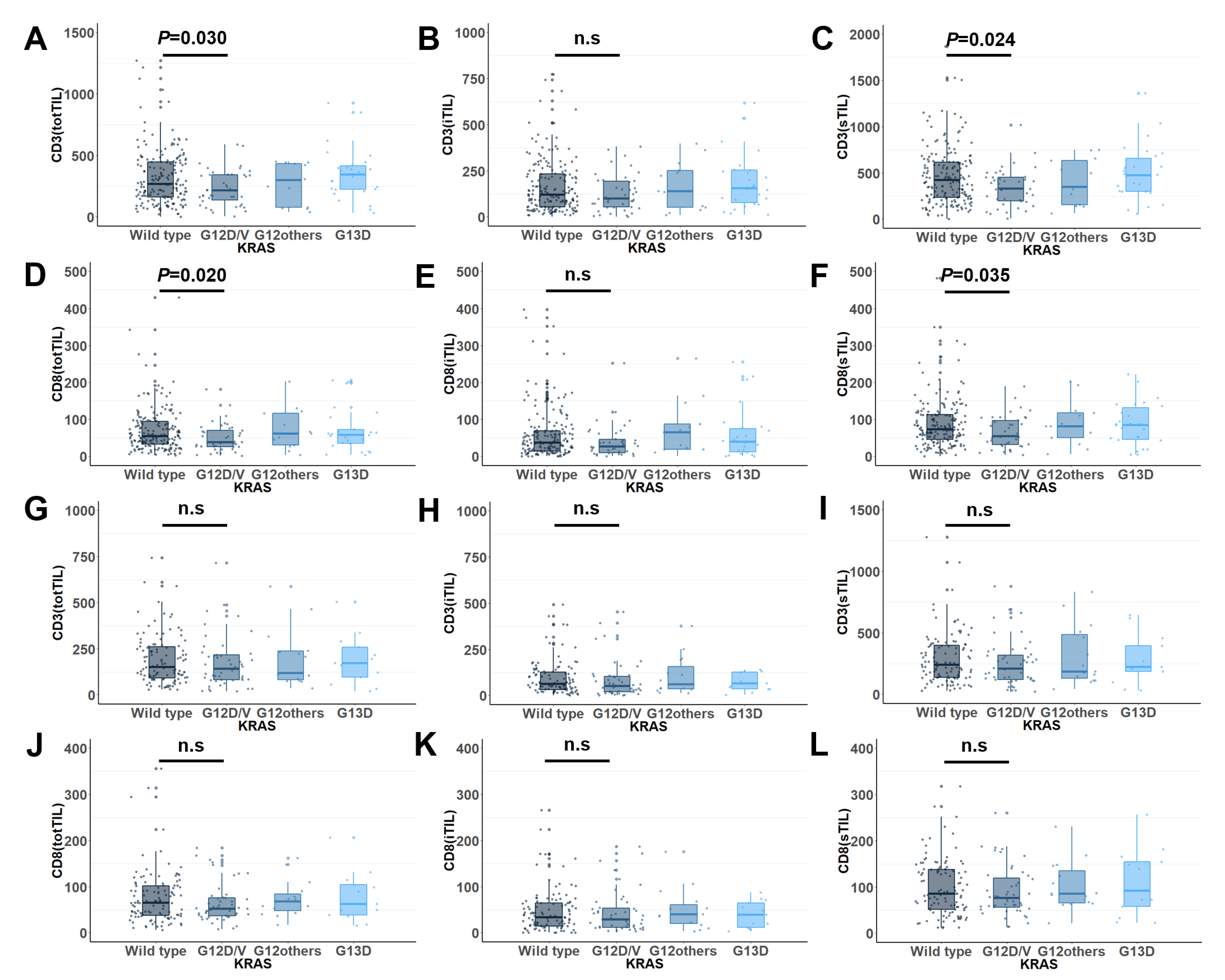

Supplement: Supplementary file 2 — Supplementary Figure 1. [file 41598_2021_94044_MOESM2_ESM.tif]
