# Supplementary material for: Tumor microenvironment-adjusted prognostic implications of the KRAS mutation subtype in patients with stage III colorectal cancer treated with adjuvant FOLFOX
Source: Sci Rep. 2021 Jul 16;11:14609. doi: 10.1038/s41598-021-94044-4 (PMC8285533; doi:10.1038/s41598-021-94044-4)
Supplement: Supplementary file 3 — Supplementary Table 1. [file 41598_2021_94044_MOESM3_ESM.docx]

**Supplementary Table 1**. Univariate survival analysis of four TIL parameters in the discovery and validation cohorts

|  | | Univariate analysis | | | |
| --- | --- | --- | --- | --- | --- |
|  | | HR (95% CI) | P-value | | |
| Discovery cohort | | | | |  |
| CD3(iTILs) |  | | |  |  |
| Low | 1 (ref) | | |  |  |
| High | 0.324 (0.183-0.575) | | | 1.16x10^-4^ |  |
| CD3(sTILs) |  | | |  |  |
| Low | 1 (ref) | | |  |  |
| High | 0.423 (0.246-0.730) | | | 1.97x10^-3^ |  |
| CD8(iTILs) | ​ | | | ​ |  |
| Low | 1 (ref)​ | | | ​ |  |
| High | 0.265 (0.146-0.483)​ | | | 1.41x10^-5^​ |  |
| CD8(sTILs) |  | | |  |  |
| Low | 1 (ref)​ | | |  |  |
| High | 0.623 (0.372-1.045) | | | 7.29x10^-2^ |  |
| Validation cohort | | | | |  |
| CD3(iTILs) |  | | |  |  |
| Low | 1 (ref) | | |  |  |
| High | 0.407 (0.185-0.894) | | | 0.025 |  |
| CD3(sTILs) |  | | |  |  |
| Low | 1 (ref) | | |  |  |
| High | 1.243 (0.598-2.584) | | | 0.560 |  |
| CD8(iTILs) | ​ | | | ​ |  |
| Low | 1 (ref)​ | | | ​ |  |
| High | 0.289 (0.124-0.677)​ | | | 0.004​ |  |
| CD8(sTILs) |  | | |  |  |
| Low | 1 (ref)​ | | |  |  |
| High | 0.784 (0.377-1.630) | | | 0.515 |  |
